# Hybrid procedure combining clip on wrapping and stent placement for ruptured supraclinoid blood blister‐like aneurysm of the internal carotid artery

**DOI:** 10.1002/ccr3.799

**Published:** 2017-02-03

**Authors:** Hirokazu Nagasaki, Michihisa Narikiyo, Gota Nagayama, Seiya Nagao, Yoshifumi Tsuboi, Chisaku Kambayashi

**Affiliations:** ^1^ Department of NeurosurgeryKawasaki Saiwai Hospital31‐27, Omiya‐cho, Saiwai‐ku, KawasakiKanagawaJapan

**Keywords:** Blood blister‐like aneurysm, stent‐assisted coil embolization, subarachnoid hemorrhage, wrap and clip

## Abstract

Blood blister‐like aneurysms of the supraclinoid portion of the internal carotid artery are rare, fragile, and thin‐walled lesions with a higher rate of rebleeding. Our case underwent a hybrid procedure combining direct surgical and endovascular approach.

## Introduction

Blood blister‐like aneurysms (BBAs) of the internal carotid artery (ICA) are rare and classically described as small, bleb‐like, broad‐necked lesions at nonbranching sites of the supraclinoid portion 1, 2, 3, 4. Pathologically, these lesions appear as focal arterial wall lacerations covered with thin fibrous tissue and adventitia lacking the usual collagen layer suggestive of a pseudoaneurysm 1, 3, 5, 6. The lesions are fragile, thin, and broad‐based and lack identifiable necks 1, 2, 3, 4. Therefore, treatment of these aneurysms is difficult, and due to a high tendency to rupture during surgery, treatment outcomes are poor, especially during the acute subarachnoid hemorrhage (SAH) period 2, 4, 6, 7.

Although several surgical approaches have been proposed for the treatment of BBAs, the optimal treatment has not been established 1, 2, 6. In the present report, we describe the case of a patient with a ruptured BBA of the ICA who underwent a hybrid procedure combining direct surgical clipping on wrapping with subsequent endovascular stent placement. To the best of our knowledge, we are the first to assess this hybrid procedure for the treatment of a ruptured BBA of the ICA. Additionally, we discuss the advantages and drawbacks of our approach and review the relevant literature.

## Case History

**Figure 1:**
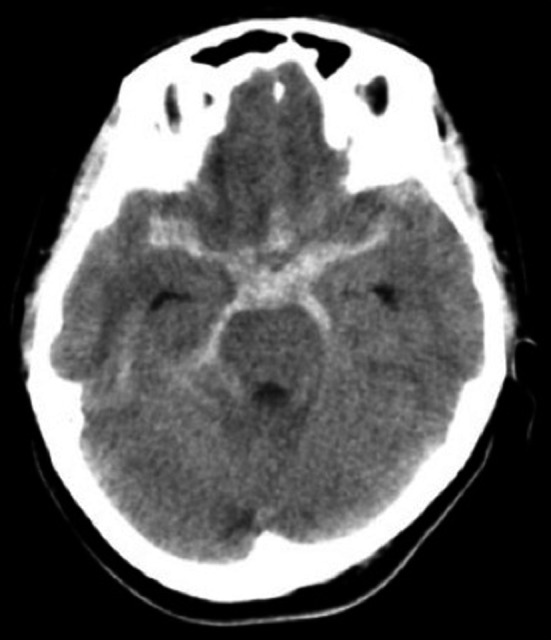
Axial computed tomography showing a diffuse subarachnoid hemorrhage in the basal cistern that is relatively prominent on the right side.

**Figure 2:**
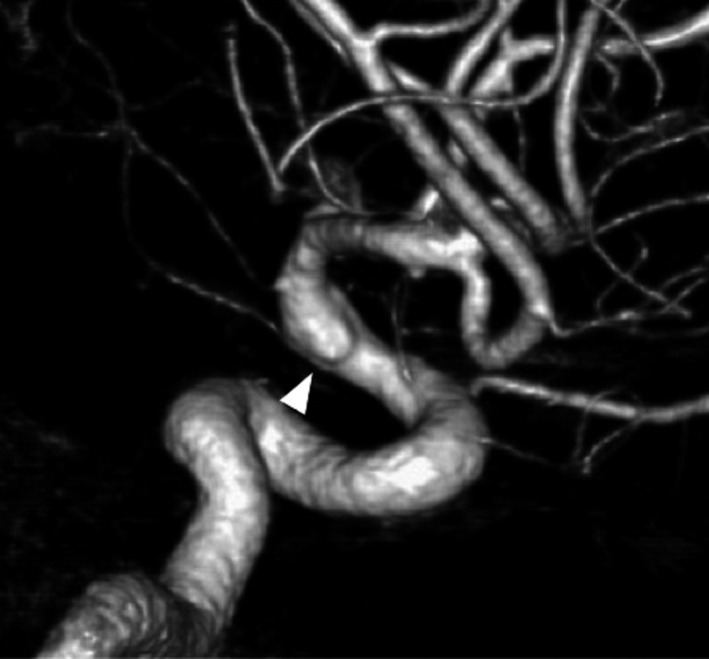
Cerebral angiography (right lateral view) revealing a subtle bump (arrowhead) on the antero‐lateral wall of the supraclinoid internal carotid artery (ICA).

**Figure 3:**
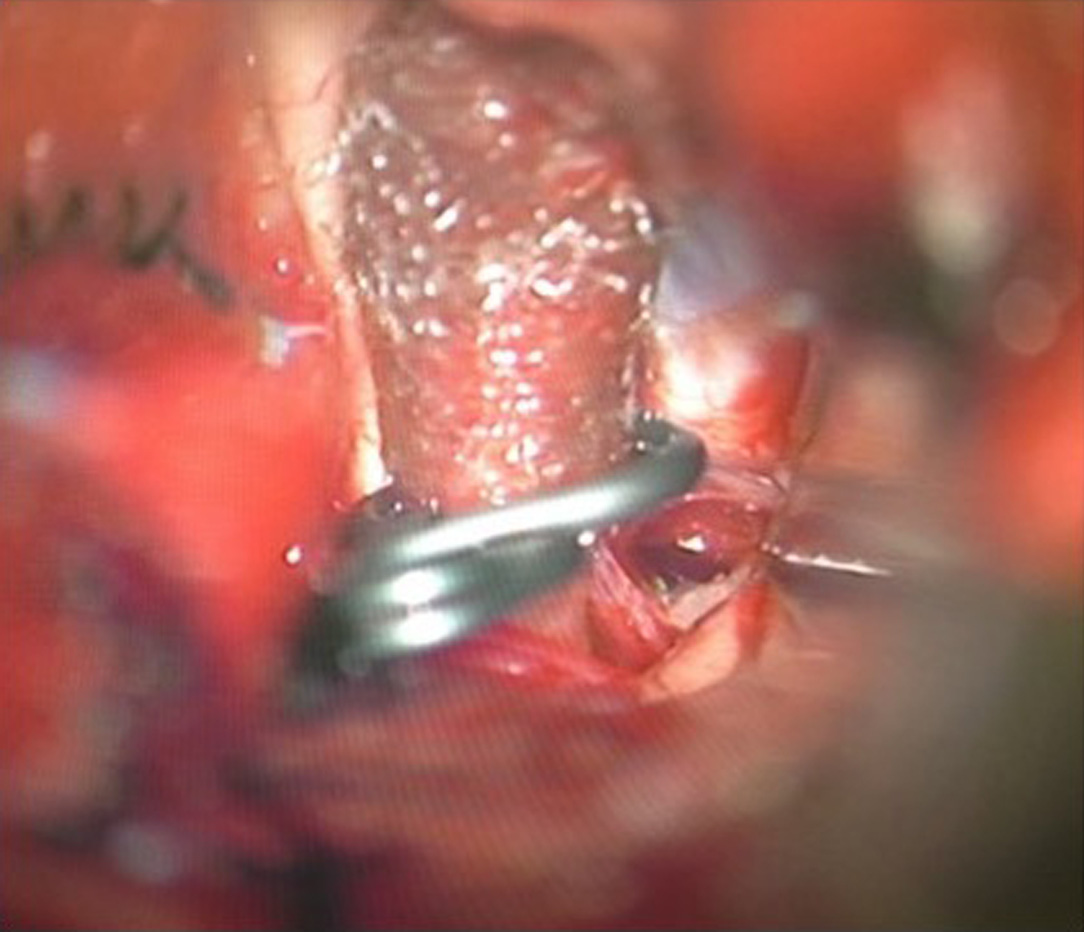
Intraoperative photograph showing clipping on wrapping of the aneurysmal segment of the internal carotid artery (ICA).

**Figure 4:**
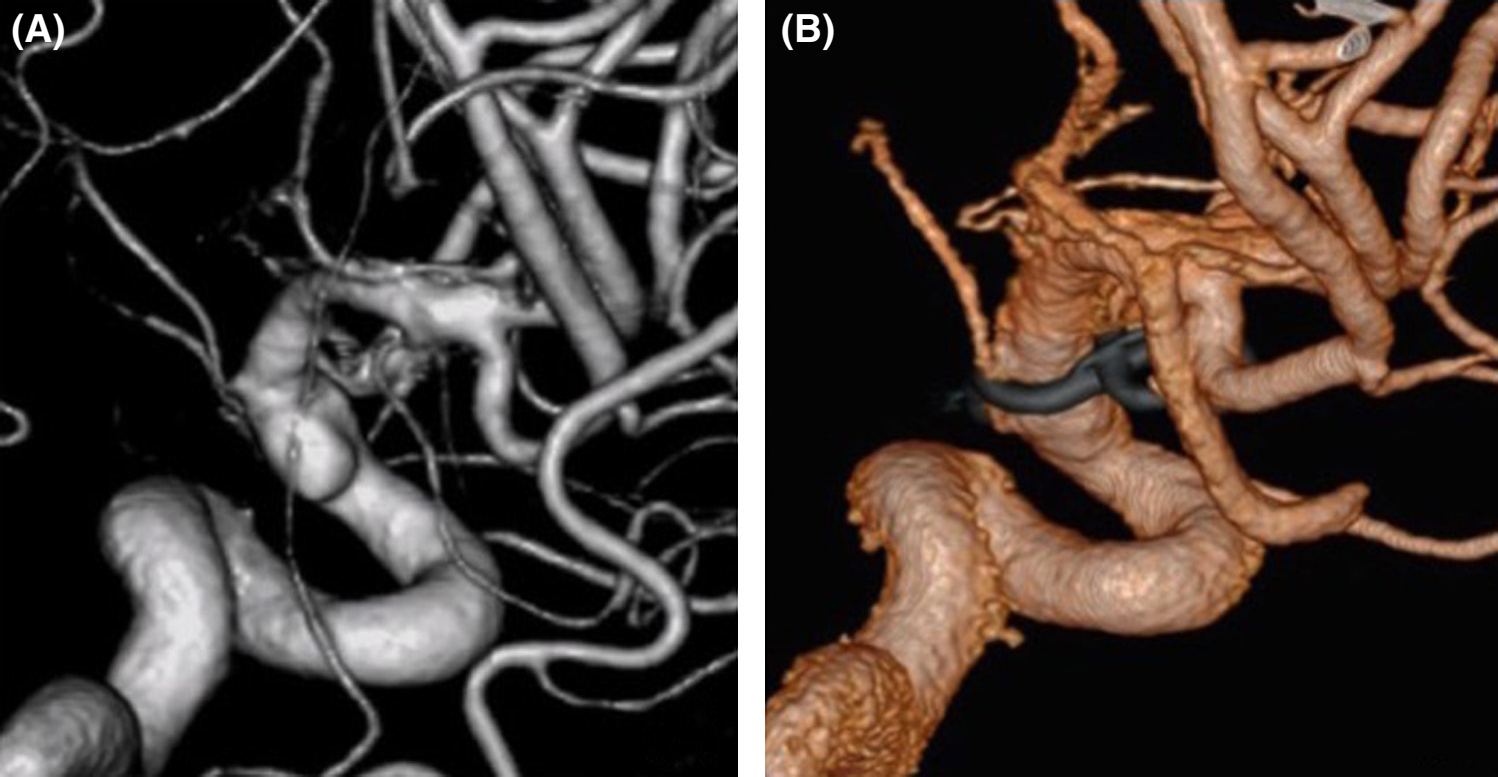
(A) Postoperative angiography revealing a residual of blood blister‐like aneurysm (BBA) on the same localization. (B) A year postoperatively, angiography demonstrating the shrinkage of the aneurysm and patency of the internal carotid artery (ICA).

**Figure 5:**
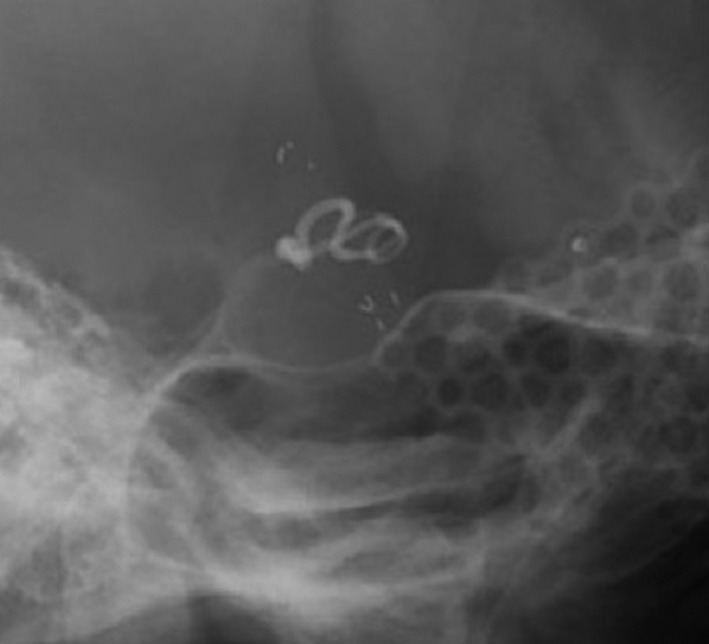
X‐ray (right lateral view) showing that the lesion is covered with encircling clip and stent.

A 42‐year‐old woman was admitted to our hospital following the sudden onset of a severe headache and vomiting. Neurological examination revealed mildly diminished consciousness and left hemiparesis, and we assigned the patient a Hunt and Hess grade of III. Computed tomography on admission showed a diffuse SAH (Fig. 1). Cerebral angiography revealed a subtle focal bump‐like irregularity of the right ICA (Fig. 2). We initially treated the patient with surgical clipping of the aneurysm to prevent rebleeding during the acute stage. A right frontotemporal craniotomy was performed revealing a discolored aneurysmal protrusion of the arterial wall, and we diagnosed this lesion as a BBA. An intradural clinoidectomy was performed because the proximal ICA exposure was inadequate. As direct placement of the clip presented the risk of aneurysm neck tearing or clip slippage, we elected to perform clipping on wrapping instead. After a temporary clip was placed on the proximal ICA, polyglycolic acid felt (Neoveil, Gunze, Ltd., Kyoto, Japan) was applied to the aneurysm and the arterial wall and held in place with fibrin glue. An encircling clip was then applied to cover the aneurysm and eliminate the space between the aneurysm and the surrounding Neoveil material (Fig. 3). The operation was uneventful. However, right oculomotor nerve palsy was noted postoperatively. Temporary compression of the oculomotor nerve during the operation may have contributed to oculomotor palsy. Postoperative angiography demonstrated a residual aneurysmal sac (Fig. 4). Therefore, we initiated a stent‐assisted coil embolization procedure and planned to insert the coils using the trans‐cell technique. Generally, two methods are used to place the microcatheter and insert the coil into the aneurysm. During the trans‐cell technique, the stent is placed first, and then, it is inserted into the microcatheter through the stent struts and into the aneurysms. During the jailing technique, the microcatheter is inserted into the aneurysms first before the stent is deployed. A self‐expanding stent (Codman Enterprise 2; Codman Neuro, Raynham, MA) was deployed to cover the aneurysm neck. We then attempted to place the coils into the aneurysmal sac; however, the aneurysm could not be coiled because of the shallow and wide‐neck morphology. Although only the stent was deployed, the patient's course was uneventful with no rebleeding, vasospasm or ischemic events. Slight oculomotor palsy remained at discharge. However, the patient recovered completely within 3 months. No neurological deficit was observed 1 year later. Follow‐up angiography and X‐ray were performed a year later (Figs. 4 and 5). The patency of the ICA is maintained, and recanalization or recurrence of the aneurysm was not observed.

## Discussion

Several surgical techniques have been advocated for the treatment of BBAs 1, 4. While the optimal surgical strategy remains controversial, there are three main approaches: direct surgery, trapping with revascularization, and endovascular surgery. Direct surgery includes aneurysm clipping, wrapping, or clipping on wrapping. Revascularization entails an external carotid artery‐internal carotid artery (EC‐IC) bypass. Endovascular approaches, such as primary coil embolization with or without stent assistance, have been described. Regardless of the treatment chosen for BBAs, each approach has risks and benefits 4.

### Direct surgery

Many consider the treatment of a BBA with surgical clipping or wrapping alone too hazardous due to the fragility of the aneurysmal wall, and these methods are often associated with poor outcome 2, 7. Clipping after placing wrapping material around the circumference of the ICA was observed to reinforce the fragile region effectively 3, 8 and may be advantageous in terms of preserving anterograde flow. Several wrapping materials and techniques have been used 8. Chronic inflammation with fibrosis and granuloma formation is a rare, delayed complication that may provoke stenosis 8.

### Trapping with revascularization

Aneurysm trapping with EC‐IC bypass includes microsurgical clip placement or endovascular coil occlusion. Theoretically, this method should prevent rebleeding and regrowth over the long term 2, 4, 7. The parent artery is sacrificed in the process of the procedure; therefore, it is impossible to retain normal arterial flow when utilizing this method. ICA trapping may result in severe infarctions when the collateral flow is insufficient 4. Based on these observations, utilizing high‐flow EC‐IC bypass with ICA trapping is suggested to provide adequate cerebral perfusion 4. While revascularization increases cerebral blood flow, whether this flow is sufficient in the event of vasospasm is still unknown, and some studies have reported that bypass flow is rarely adequate protection against ischemia if vasospasm occurs 1.

Endovascular treatment for vasospasm is technically challenging after ICA trapping. Because the ipsilateral carotid artery is occluded, the microcatheter should be placed through the contralateral ICA 7. Graft occlusion may be an additional risk in the long term 5. The position of the lesion is also a consideration, and parent artery occlusion is not suitable for cases in which the anterior choroidal (Ach) or posterior communicating (Pcom) artery arises close to the BBA 1, 9.

### Endovascular treatment

As BBAs are believed to be pseudoaneurysms, endovascular coil embolization alone should not be considered a first‐line treatment 5, 6. Stent assistance, as an adjunct procedure, has been reported to improve success 6. However, stent‐assisted coil embolization may result in high residual and recurrence rates 5. The overlapping stent technique is an alternative to the use of a single stent that may decrease aneurysm inflow and promote thrombosis of the BBA more effectively 9. On the other hand, subsequent treatment necessitated by residual or recurrent disease could be rendered more difficult due to the stent strut density and thickness 6. Furthermore, the optimal antithrombotic regimen for patients undergoing endovascular stenting in the acute SAH phase remains uncertain due to the extreme fragility of these lesions 6, 9. The other concern is the long‐term patency of the parent artery managed with endovascular stenting 9.

Recently, flow diverter stents have been developed. These stents divert blood flow away from the aneurysm and may allow reconstruction of the parent artery. Long‐term clinical and angiographic surveillance of patients treated with flow diverting stents is recommended.

Covered stents are another option. However, they are far stiffer and may carry the risk of additional damage to the vessels 4. In addition, BBAs are often located in proximity to the origins of the Ach and Pcom arteries, making the use of a covered stent less suitable 6.

In our case, we initially performed clip on wrapping followed by stent placement, a technique that reinforces the inside and outside of the aneurysm. Full circumferential wrapping of a BBA could help reduce the risk of intraoperative rebleeding during coiling by providing external reinforcement and may also reduce the rates of residual and recurrent disease. Stenosis occurring as a complication of the wrapping material may be minimized in this case by the presence of a self‐expanding stent. Flow diverter stents can also be used as an alternative treatment. In Japan, flow diverter stents (Pipeline Embolization Device, Medtronic, Irvine, CA) can be used for limited indications. The pipeline embolization device is indicated for unruptured aneurysms with a diameter of 10 mm or larger in the internal carotid artery from the petrous to the superior hypophyseal segments. Moreover, deploying the Enterprise stent is technically easier than deploying flow diverter stents. Therefore, we elected to use Enterprise stents, because we could deploy the stents precisely and overlap them.

While trapping with revascularization reliably avoids rebleeding, we prefer to preserve the parent artery and maintain normal arterial flow as this facilitates management of vasospasm in the acute SAH phase. Furthermore, BBA is located close to the Ach and Pcom arteries in the present case. Hence trapping was not elected.

Management planning for patients presenting with a BBA must be tailored to the clinical and surgical conditions, and each available procedure has benefits and drawbacks. A hybrid procedure may minimize the disadvantages presented by any single treatment method and could be considered a definitive treatment for ruptured BBAs.

## Conflict of Interest

None declared.

## Authorship

HN: designed the study and wrote the initial draft of the manuscript. GN and MN: assisted in the preparation of the manuscript. SN and YT: contributed to upgrade the content. CK: critically reviewed the manuscript.
